# Chinese public’s knowledge, perceived severity, and perceived controllability of COVID-19 and their associations with emotional and behavioural reactions, social participation, and precautionary behaviour: a national survey

**DOI:** 10.1186/s12889-020-09695-1

**Published:** 2020-10-21

**Authors:** Jian-Bin Li, An Yang, Kai Dou, Lin-Xin Wang, Ming-Chen Zhang, Xiao-Qi Lin

**Affiliations:** 1 Department of Early Childhood Education, The Education University of Hong Kong, Hong Kong Special Administrative Region, P. R. China; 2 Department of Applied Psychology, Guangdong University of Foreign Studies, Guangzhou, P. R. China; 3 Department of Psychology and Research Center of Adolescent Psychology and Behavior, School of Education, Guangzhou University, 230, Waihuan Road West, Panyu District, Guangzhou, P. R. China

**Keywords:** COVID-19, Cognitive appraisal, Psychological health, Social participation, Precautionary behaviour

## Abstract

**Background:**

The outbreak of the coronavirus disease-19 (COVID-19) has caused enormous stress among the public in China. Intellectual input from various aspects is needed to fight against COVID-19, including understanding of the public’s emotion and behaviour and their antecedents from the psychological perspectives. Drawing upon the cognitive appraisal theory, this study examined three cognitive appraisals (i.e., perceived severity, perceived controllability, and knowledge of COVID-19) and their associations with a wide range of emotional and behavioural outcomes among the Chinese public.

**Methods:**

Participants were 4607 citizens (age range: 17–90 years, Mage = 23.71 years) from 31 provinces in China and they took part in a cross-sectional survey online.

**Results:**

The results showed that the public’s emotional and behavioural reactions were slightly affected by the outbreak of COVID-19. Moreover, the public had limited participation in the events regarding COVID-19 but actively engaged in precautionary behaviour. In addition, results of structural equation model with latent variables revealed that the three appraisals were differentially related to the outcome variables (i.e., negative emotion, positive emotion, sleep problems, aggression, substance use, mobile phone use, social participation, and precautionary behaviour).

**Conclusions:**

The findings highlight the utility of cognitive appraisal, as a core process of coping stress, in explaining the public’s emotion and behaviour in the encounter of public health concern. Practically, the findings facilitate the government and practitioners to design and deliver targeted intervention programs to the public.

## Background

In December 2019, several cases of pneumonia with unknown causes were reported in Wuhan, the capital of Hubei Province in central China. The pneumonia was later diagnosed to be caused by a novel coronavirus and named Coronavirus Disease-19 (COVID-19) by the World Health Organization [1]. Since then, COVID-19 has broken out from Hubei Province, particularly from Wuhan city, and spread across mainland China rapidly. Later, COVID-19 has spread outside China, posing risks to other countries. At the end of January 2020, The World Health Organization has declared the outbreak of COVID-19 in China a Public Health Emergency of International Concern [2]. At the beginning of February 2020 right before the current survey commenced, COVID-19 has caused thousands of diagnosed cases and hundreds of deaths. The Chinese government has actively adopted a variety of measures to control COVID-19, including implementing effective medical treatment, monitoring the progress, issuing factsheets and precautionary guidelines, and even controlling the mobility of the population within the city and between cities. However, citizens received the information about COVID-19 from various channels and might have different knowledge about COVID-19. In addition, the numbers of suspicious and diagnosed cases and mortality were still increasing in most provinces before this study was conducted, which also affected the public’s perception about the severity and controllability of COVID-19. Individuals with different knowledge and perception of COVID-19 could show different emotional and behavioural reactions towards COVID-19.

A timely understanding of the public’s knowledge and perception of COVID-19 as well as their associations with individuals’ emotion and behaviour was still lacking before the implementation of this study in early February 2020. Public’s knowledge, perception, precautionary behaviour and active social participation have been found to be important in the control of epidemics, as learned regarding severe acute respiratory syndrome (SARS), Ebola, and H1N1 [3–5]. Nevertheless, every public health concern occurs at different periods in different places and each country/region possesses different magnitudes of resources to reduce the detriment it brings. In this sense, there could be both commonalities and variations in the emotional and behavioural reactions caused by different events of public health concern. Therefore, it is necessary to investigate the public’s emotional and behavioural outcomes and their antecedents in the encounter of COVID-19. Hopefully, the findings may deepen the understanding of the public’s appraisal processes in the encounter of emergent public health concern and provide early evidence to relevant stakeholders, policy-makers, and practitioners to better develop and deliver tailor-made psychological aids to the public affected by COVID-19. In sum, drawing upon the proposition that cognitive appraisal as a process of coping stress [6, 7], the present research aims to examine the public’s perceived severity, perceived controllability, and knowledge, of COVID-19 and their associations with emotional and behavioural reactions, social participation, and precautionary behaviour.

### Cognitive appraisal and emotional and behavioural reactions

Studies on SARS have indicated that the outbreak of an unprecedented virus can cause immense stress to the public of different age, professionals, and regions [8–11]. Encounter of environmental stress may induce individuals to use different methods to cope with the stress and maintain their health and well-being. Cognitive appraisal, as a core process in coping stress, is supposed to closely associate with immediate and long-term outcomes [12].

Cognitive appraisal is a process through which the person evaluates whether a particular encounter with the environment is relevant to his or her well-being, and if so, in what ways [12]. It consists of two stages. *Primary appraisal* refers to a person’s estimate of whether he or she has anything at stake in the encounter [12]. Encounters can be evaluated as irrelevant, benign-positive (beneficial) or stressful [13]. For instance, is the encounter potentially harmful or beneficial to a person’s well-being or health? Assessment of a person’s evaluation of what is at stake in the outcome of the encounter is a critical indicator of the primary appraisal, such as assessing how disturbing, threatening or challenging of the encounter is [13, 14]. *Secondary appraisal* refers to a person’s evaluation of what can be done to overcome/prevent harm or to increase the benefit; this process involves a complex assessment of a person’s coping options [12]. Evaluation of the extent to which a situation requires more information and how controllable the situation is has been regarded as crucial indicators of the secondary appraisal [13].

Prior research has associated indicators of primary and secondary appraisals with a wide range of emotional and behavioural outcomes. For example, Peacock and Wong found that when individuals perceive an encounter to be more threatening, uncontrollable and stressful, they reported higher levels of psychological symptoms and dysphoric mood [13]. Oliver and Brough revealed that perceived controllability was predictive of individuals’ well-being [15]. In another research, Gomes, Faria, and Lopes found that perceptions of threat, control, and challenge of stressful encounters were significantly related to mental health problems [16]. Besides, cognitive appraisal has also been applied to the study of the public’s emotion and behaviour during the outbreak of emergent public health concerns. For instance, in Dorfan and Woody’s study, they measured individuals’ appraisal of danger, germ spread and responsibility and associated them with a number of emotion and behaviour towards the outbreak and transmission of SARS, including avoidance, disgust, anxiety, urge to wash, washing duration, and wipes taken [3]. Their findings disclosed that appraisal of danger of SARS was significantly related to emotional and behavioural responses. Another research found that knowledge and perception of SARS were related to precautionary behaviour [17]. In addition, Yang and Chu found that perceived risk of virus was related to higher levels of negative emotion (i.e., fear, anxiety, disgust, and anger) about the outbreak of Ebola in the U.S. public [4].

Some studies have directly examined cognitive appraisal factors and emotional and behavioural reactions during the outbreak of COVID-19. For instance, prior studies have conducted descriptive research to examine the levels of knowledge, attitude, preventive behaviour and risk perception regarding COVID-19, revealing that most participants reported high levels of knowledge of COVID-19 and had strong intention to engage in preventive behaviour [18, 19]. Some studies have also carried out epidemiological survey about individuals’ mental health, psychological distress, and well-being during the outbreak of COVID-19, revealing that most citizens reported substantial mental health problems and [20, 21]. Besides, some studies have also disclosed that perceiving COVID-19 to be severe was associated with more mental health problems (e.g., depressive symptoms and anxiety) [22–24], and more preventive behaviour [25]. In addition, prior research also found that high levels of knowledge were related to stronger intention to follow and actual engagement in preventive behaviour [25, 26]. However, there were also a few inconsistent findings which revealed that more knowledge about COVID-19 and perceiving COVID-19 to be severe were associated with *less* preventive behaviour [27, 28].

Based on the existing literature reviewed above, we learn that most studies that examined the cognitive appraisal factors regarding COVID-19 primarily focused on knowledge and perceived severity of COVID-19 and that most studies examined these cognitive appraisal factors separately. From a theoretical aspect, cognitive appraisal includes both primary (e.g., perceived severity) and secondary (e.g., perceived controllability) appraisal. However, scant research has investigated the role of perceived controllability, a crucial secondary appraisal factors in stress coping, in the emotional and behavioural outcomes towards the outbreak of COVID-19. From a methodological aspect, different types of cognitive appraisal factors could be overlapped and the association between a single cognitive appraisal factor and the outcomes could be inflated without considering them simultaneously. This study, although conducted at the early stage of the outbreak of COVID-19 in early February 2020, contributes to the literature in that it examines three types of cognitive appraisal factors (i.e., perceived severity, knowledge, and perceived controllability) at the same time, which allows us to control for the covariance between these factors and thus we may obtain a more nuanced estimation of the associations between these appraisal factors and the outcomes. In addition, we also examine some more outcomes that have received comparatively less attention in past studies, such as social participation and mobile phone use. In sum, the findings of this study are expected to shed light on the mitigation of negative and the promotion of positive emotional and behavioural reactions during the outbreak of COVID-19.

### The current study

Drawing upon the cognitive appraisal theory, this study aims to understand the public’s perceived severity, perceived controllability, and knowledge of COVID-19 and their associations with emotional and behavioural reactions, social participation, and precautionary behaviour. In this study, we consider the public’s perceived severity as the primary appraisal, as it relates to an individual’s evaluation of how likely their health and well-being is at stake in the encounter of COVID-19, and we consider the public’s perceived controllability and knowledge of COVID-19 as secondary appraisal, as they reflect as the intellectual and mental resources to cope with the stress and disturbance caused by COVID-19. In light of the literature reviewed above, these cognitive appraisals are supposed to be related to a number of emotional and behavioural reactions. Since the numbers of diagnosed cases and the rate of mortality of COVID-19 differ greatly among different regions in China, the public’s emotional and behavioural responses, social participation, and precautionary behaviour could vary as well. To take this into account, we recruited sample from a wide array of provinces in mainland China. We took into account a number of demographic variables as well to control for their potential effects on the outcomes.

## Method

### Design

A cross-sectional design survey with national sample was conducted.

### Participants and procedure

A snowball sampling was used to recruit participants from different regions in China. A total of 4826 Chinese visited the online survey between 2 February and 9 February, 2020. Sixty-nine participants were not willing to participate in the study, leaving 4757 participants to take part in the survey, resulting in a response rate of 98.6%. Then, we removed a number of participants because (1) they indicated that they were under 16 years old (*N* = 99), a cutting age that parent consent is optional, or (2) they were inclined to respond to the items in a similar pattern (e.g., chose the same answer across multiple consecutive items or within the whole questionnaire, *N* = 51). Finally, 4607 participants provided complete data^1^ and were included in the analyses. Participants’ age ranged from 17 to 90 years old (Mean age = 23.71 years old, SD = 7.29). They were from 31 provinces / centrally-governed cities / autonomous regions / special administrative regions, with the sample size ranging from 16 (0.3% of the total sample, Ningxia Hui Autonomous Region) to 1386 (30.1% of the total sample, Guangdong Province). The sample covered a wide range of demographics. Detailed demographics are summarized in Table 1.

**Table 1:** Summary of demographic variables

	N	%
**Biological sex**		
Male	1265	27.5
Female	3342	72.5
**Educational level**		
Junior middle school and below	79	1.7
High school degree	343	7.4
College degree	810	17.6
Bachelor degree	3129	67.9
Master degree	217	4.7
Doctoral degree	29	.6
**Current residential provinces**		
Anhui Province	152	3.3
Beijing	33	.7
Chongqing	28	.6
Fujian Province	223	4.8
Gansu Province	31	.7
Guangdong Province	1386	30.1
Guangxi Zhuang Autonomous Region	74	1.6
Guizhou Province	110	2.4
Hainan Province	20	.4
Hebei Province	58	1.3
Henan Province	108	2.3
Heilongjiang Province	40	.9
Hong Kong Special Administrative Region	82	1.8
Hubei Province	45	1.0
Hunan Province	383	8.3
Inner Mongolia Autonomous Region	18	.4
Jilin Province	30	.7
Jiangsu Province	124	2.7
Jiangxi Province	1005	21.8
Liaoning Province	28	.6
Ningxia Hui Autonomous Region	16	.3
Qinghai Province	92	2.0
Shandong Province	101	2.2
Shanxi Province	57	1.2
Shaanxi Province	35	.8
Shanghai	50	1.1
Sichuan Province	62	1.3
Tianjin	42	.9
Xinjiang Uygur Autonomous region	42	.9
Yunnan Province	50	1.1
Zhejiang Province	82	1.8
**Self-reported current physical health condition**		
Very poor	5	.1
Poor	46	1.0
Average	997	21.6
Good	2160	46.9
Very good	1399	30.4
**Self-reported history of chronic physical diseases**		
Yes	251	5.4
No	4356	94.6
**Self-reported history of psychiatric / psychological disorder**		
Yes	39	.8
No	4568	99.2
**Relationship with COVID-19**		
Healthy (not infected)	4499	97.7
Other	108	2.3
**Total**	4607	100

The study was reviewed and approved by Guangzhou University. The whole study was conducted online in compliance with the ethical standards for research outlined in the Ethical Principles of Psychologists and Code of Conduct [29]. Over 200 student helpers who majored in psychology voluntarily distributed the online survey link on various internet platforms, including WeChat (the most popular APP for instant message in mainland China), Weibo, QQ, etc. By clicking the hyperlink, participants were directed to an online survey website. An information sheet stating the goal and the procedure of the study was presented to participants on the first page of the survey. If participants checked the “I understood the study and am willing to participate” box at the bottom of the information sheet, they would entered the survey and fill in the questionnaires. If participants were not willing to participate, they could check the “I understood the study but am not willing to participate” box and then the survey ended. Participation was voluntary and no incentive reward was given. Anonymity was emphasized and no identifiable information was collected. It took participants about 20 min to complete the survey.

### Measures^2^

#### Emotional and behavioural reactions

Participants’ emotional and behavioural reactions were measured with 20 items. These items cover a number of dimensions, including negative emotion (8 items, anxiety, worry, depressive, panic, lonely, nervous, sad, and angry), positive emotion (3 items, happy, joy, and excited), sleep problems (4 items, insomnia, shallow sleep, have nightmares, and insufficient sleep), aggression (2 items, argue with others and physical fight with others), substance use (2 items, smoking and drinking), and mobile phone use (1 item). Participants were asked to indicate the differences in the emotional and behavioural reactions listed above before and after the outbreak of COVID-19 on a five-point scale (from “1 = *much less compared to the days before the outbreak*” to “5 = *much more compared to the days before the outbreak*”). To align with other dimensions, the items for positive emotion were *reversely* scored. A higher score indicates COVID-19 causes *more* negative emotion, sleep problems, aggression, substance use, mobile phone use, and *less* positive emotion.

#### Social participation

The Social Participation Scale used in prior research [30] was adapted to measure participants’ social participation regarding COVID-19. Participants were asked to indicate how often they participated in different social events since the outbreak of COVID-19 on a five-point scale (from “0 = *never*” to “4 = *very often*). A higher score indicates that participants participated in the social events more actively. A sample item is “How often do you help those who need help in the community since the outbreak of COVID-19?”

#### Precautionary behaviour

Participants’ precautionary behaviour was measured with 19 items written by authors following the precautionary guideline issued by the Chinese government. Participants were asked to indicate how often they show various precautionary behaviour since the outbreak of COVID-19 on a five-point scale (from “0 = *never*” to “4 = *very often*). A higher score indicates that participants comply with precautionary behaviour more frequently. Sample items include *avoiding travelling to regions affected by COVID-19*, *wearing a facemask*, *regularly changing a facemask*, and *washing hands*.

#### Knowledge about COVID-19

Participants’ perceived knowing of various aspects of COVID-19 (e.g., cause, ways of transmission, symptoms, diagnostic criteria, etc.) was measured with 11 items. Participants indicated how much they know each item on a five-point scale (from “1 = *totally not know*” to “5 = *totally know*”). A higher score indicates participants perceived they know more about COVID-19.

#### Perceived severity

Participants’ perceived severity about COVID-19 was measured with 5 items. Participants indicated their perception of how severe is the infection rate, morbidity, mortality, the negative influence on social order and the negative influence on the economics on a five-point scale (from “1 = *not severe at all*” to “5 = *very much severe*”). A higher score indicates participants perceived COVID-19 to be more severe.

#### Perceived controllability

Participants’ estimation of how much can the various aspects of COVID-19 be controlled was measured with 9 items on a five-point scale (from “1 = *totally uncontrollable*” to “5 = *totally controllable*”). A higher score indicates participants perceived COVID-19 to be more controllable. A sample item is “How controllable do you think the etiology of COVID-19 is?”

#### Demographic variables

We also collected a number of demographic variables of participants, including their biological sex (0 = *male*, 1 = *female*), age, education (1 = *junior middle school or below*, 2 = *high school or equivalent*, 3 = *college*, 4 = *bachelor degree*, 5 = *master degree*, 6 = *doctoral degree*), current residential location (referred to province and city/district), their relationship with COVID-19 (1 = *healthy*, 2 = *other, including suspicious case, diagnosed case, relatives or friends of suspicious/diagnosed case,* etc.), the history of chronic physical diseases and psychiatric/psychological disorder (1 = *yes*, 2 = *no*), and their current physical health condition (from “1 = *very poor*” to “5 = *very good*”).

### Data analysis

We analysed the data in SPSS and Mplus 7.0, with .05 as the significant level across all analyses. We conducted preliminary analyses prior to carrying out formal statistical analyses. First, we examined the psychometric properties of the measures used in this study, including internal consistency reliability, item discrimination and confirmatory factor analysis. Second, given that only self-report questionnaires were used in this study and this might cause common method variance, we examined whether common method variance should be of a concern in this study. Subsequently, we then continued performing formal analyses. First, we conducted the descriptive statistics to capture the centrality of the variables. Second, we conducted correlation analysis to capture the association between participants’ knowledge about COVID-19, perceived severity, and perceived controllability and emotional and behavioural reactions, social participation, and precautionary behaviour. For the correlation analysis, we employed Cohen’s (1992) standard to determine whether the correlation coefficients were substantial, with *r* = .01, .03, and .05 representing small, medium, and large effect size [31]. Last, given that the data structure is hierarchical in nature (i.e., participants nested in provinces), multilevel regression analysis should be used. Prior to using multilevel model, we examined the intraclass correlation (ICC) for each outcome variable. We found that the ICCs were trivial, ranging from .008 (sleep problem) to .032 (negative emotion). Given that the ICCs were smaller than .05, we believed that treating the data as individual data would be appropriate and thus would not choose multilevel regression model. We employed Mplus 7.0 to conduct regression analysis with latent variables. We used the items from each measure to construct the latent variables except for perceived controllability, knowledge about COVID-19, negative emotions, and precautionary behaviour because these four measures had a number of items, which may render poor model fit. To construct latent variables for these four measures, we used an item-to-construct balance parceling technique [32] to create three indicators for each measure. We controlled for a number of demographic variables when we examined the associations between independent variables and the dependent variables. The values of RMSEA (< .08), CFI and TLI (> .90) indicate the model fit is acceptable [33, 34].

## Results

### Preliminary analyses

We conducted a number of tests to examine the reliability and the validity of the measures used in this study, and the results are summarized in Table 2. First, the internal consistency reliabilities of each measure/dimension were good, ranging from .79 to .97. Second, we performed item discrimination tests by comparing the differences in items between groups with high (top 27%) and low (bottom 27%) total score in each measure/dimension. For instance, the “Knowledge about COVID-19” measure consists of 11 items. We examined whether these 11 items were significantly different between groups with high and low total score of this measure. The range of t-value (e.g., [− 63.90, 45.57]) represented individual *t*-test values for the 11 items. The results showed that all the items in each measure/dimension had good ability to differentiate groups with high and low total score of that measure/dimension. Third, we carried out confirmatory factor analyses for each measure/dimension which had at least 3 items. We examined the one-factor model for each measure/dimension. With some residuals correlated for some measures/dimensions, the results showed that all the tested models had acceptable model fit (CFI > .90, RMSEA & SRMR < .08.). Taken together, these tests suggested that the measures used in this study could be seen as reliable and valid. Moreover, to examine whether common method variance should be of a concern in this study, we carried out an exploratory factor analysis with all the measurement items (i.e., Harman’s single-factor test). The results showed that the primary factor component only accounted for about 16.40% variance, which suggested that common method bias should not be a concern in this study.

**Table 2:** Psychometric properties of the measures used in this study

	Reliability	Item discrimination of each measure ^a^		Model fit of confirmatory factor analysis (CFA) ^d^					
	*α*	*t* ^*b*^	*p*	*χ*^2^	*df*	CFI	RMSEA	90%CI	SRMR
**1. Knowledge about the COVID-19**	0.91	[−63.90, −45.57]	< 0.001	386.87	24	0.99	0.06	[0.052, 0.062]	0.02
**2. Perceived severity**	0.84	[−65.61, −52.08]	< 0.001	62.06	3	1.00	0.07	[0.052, 0.080]	0.02
**3. Perceived controllability**	0.91	[−93.59, −49.78]	< 0.001	135.78	17	1.00	0.04	[0.033, 0.045]	0.01
**4. Emotional and behavioural reactions**									
Negative emotion	0.91	[−59.46, −34.28]	< 0.001	192.48	8	0.99	0.07	[0.062, 0.080]	0.02
Positive emotion	0.97	[− 115.87, − 114.46]	< 0.001	e	e	e	e	e	e
Sleep problem	0.91	[−42.78, −29.82]	< 0.001	24.11	2	0.99	0.05	[0.033, 0.067]	0.01
Aggression	0.90	[−60.09, −22.67]	< 0.001	f	f	f	f	f	f
Substance use	0.91	[−27.75, −15.85]	< 0.001	f	f	f	f	f	f
Mobile phone use	–	−101.18 ^c^	< 0.001	f	f	f	f	f	f
**5. Social participation**	0.79	[− 70.45, −47.41]	< 0.001	23.71	3	1.00	0.04	[0.025, 0.054]	0.01
**6. Precautionary behaviour**	0.92	[−55.19, −33.11]	< 0.001	2950.96	121	0.96	0.07	[0.069, 0.073]	0.08

^a^: The ability of each item in a measure/dimension to differentiate the high (top 27%) and the low (bottom 27%) total score of that measure/dimension was examined

^b^: T-values of the comparison between the high and the low total score groups in the item discrimination tests are presented as a range. For instance, the measure of “Knowledge about the COVID-19” consists of 11 items; the range represents the *t* values of the comparison for these 11 items

^c^: The “mobile phone use” dimension has only 1 item and thus no range is presented

^d^: Some residuals are correlated when we fit the CFA models for some measures/dimensions

^e^: This dimension has 3 items and thus the CFA model is a saturated model (i.e., *χ*^2^ = 0.00, *df* = 0.00, CFI = 1.00, RMSEA and SRMR = 0.00)

^f^: Each of these dimensions has less than 3 items and thus CFA is not applicable

### Descriptive statistics

As shown in Table 3, participants indicated that they had medium level of knowledge about COVID-19 (3.56 out of 5). Moreover, participants perceived COVID-19 to be highly severe (4.09 out of 5) and modestly controllable (3.25 out of 5). Regarding their emotional and behavioural reactions, the results showed that COVID-19 did not change much of the frequency of participants’ positive and negative feelings and a range of behaviour, with the mean score ranging from 2.61 to 3.77. In fact, participants indicated that the frequencies of sleep problem, aggression, and substance use after the outbreak were slightly lower compared to the ones before the outbreak of COVID-19. As for social participation, participants appeared not to actively participate in the social events regarding COVID-19 (1.75 out of 4). However, participants reported that they displayed intensive precautionary behaviour to prevent COVID-19 (3.33 out of 4).

**Table 3:** Descriptive Statistics of knowledge about the COVID-19, perceived severity, perceived controllability, emotional and behavioural reactions, social participation, and precautionary behaviour

	*Number of Items*	*Possible range*	*M*	*SD*	*Skewness*
**1. Knowledge about the COVID-19**	11	1–5	3.56	.61	−0.35
**2. Perceived severity**	5	1–5	4.09	.59	−0.82
**3. Perceived controllability**	9	1–5	3.25	.72	−0.20
**4. Emotional and behavioural reactions**					
Negative emotion	8	1–5	3.33	.67	−1.11
Positive emotion	3	1–5	3.68	.83	0.12
Sleep problem	4	1–5	2.79	.76	−1.12
Aggression	2	1–5	2.70	.78	−1.25
Substance use	2	1–5	2.61	.80	−1.19
Mobile phone use	1	1–5	3.77	.97	−0.54
**5. Social participation**	5	0–4	1.75	.77	0.24
**6. Precautionary behaviour**	19	0–4	3.33	.66	−1.43

### Associations between the variables of interest

The bivariate correlation coefficients of the association between the variables of interest are summarized in Table 4. The results showed that participants’ knowledge about COVID-19 was positively related to social participation and precautionary behaviour. Participants’ perceived severity was positively related to the increase in negative emotion and mobile phone use, decrease in positive emotion, and more precautionary behaviour. Although the associations between perceived severity and the changes in sleep problems and social participation were also significant, the effect sizes were too trivial to explain (i.e., *r* < .10). Finally, perceived controllability was negatively related to the increase in negative emotion and more social participation and precautionary behaviour. Although the associations between perceived controllability and the changes in positive emotion, sleep problems, and mobile phone use were also significant, the effect sizes were too small to explain (i.e., *r* < .10).

**Table 4:** Bivariate Correlations of knowledge about the COVID-19, perceived severity, perceived controllability, emotional and behavioural reactions, social participation, and precautionary behaviour

Variables	1	2	3	4	5	6	7	8	9	10	11
**1. Neg.**	–										
**2. Pos.**	.15^***^	–									
**3. Sleep.**	.44^***^	−.20^***^	–								
**4. Agg.**	.38^***^	−.24^***^	.70^***^	–							
**5. SU**	.33^***^	−.28^***^	.67^***^	.79^***^	–						
**6. MPU**	.38^***^	.10^***^	.23^***^	.17^***^	.12^***^	–					
**7. Soc. P.**	.11^***^	.03	.04^***^	.00	.00	.03^*^	–				
**8. Pre. B**	.10^***^	.04^*^	.00	.00	.01	.11^***^	.25^***^	–			
**9. Know**	.00	−.02	.00	−.01	.00	.03	.24^***^	.30^***^	–		
**10. PerS**	.24^***^	.15^***^	.06^***^	.01	−.03	.20^***^	.09^***^	.27^***^	.13^***^	–	
**11. PerC**	−.10^***^	−.09^***^	−.04^**^	−.02	.00	−.04^*^	.10^***^	.15^***^	.37^***^	−.09^***^	–

*Neg.* negative emotion, *Pos.* positive emotion, *Sleep* sleep problems, *Agg.* aggression, *SU* substance use, *MPU* mobile phone use, *Soc. P.* social participation, *Pre. B* precautionary behaviour, *Know* knowledge about the COVID-19, *PerS* perceived severity, *PerC* perceived controllability

*p < .05; ** *p* < .01; *** *p* < .001

### The associations of knowledge, perceived severity, and perceived controllability with emotional and behavioural reactions, social participation, and precautionary behaviour

**Fig. 1:**
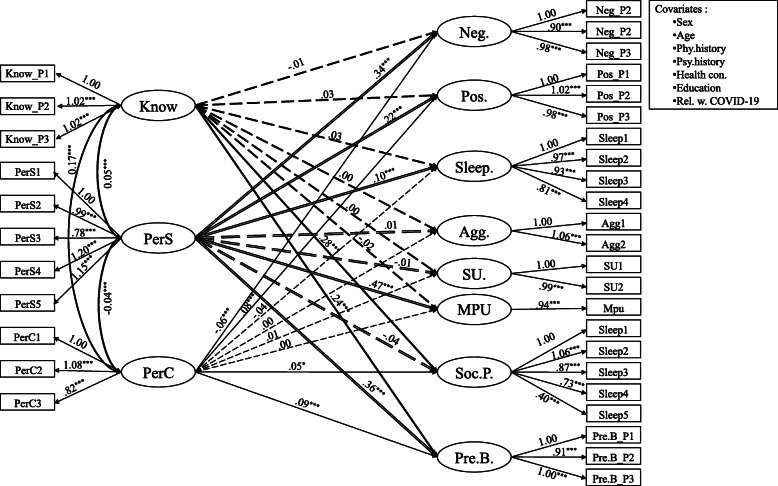
The association between perceives severity, controllability and knowledge of COVID-19 and emotional and behavioural reactions, social participation, and precautionary behaviour. *Note*. Know = knowledge about the COVID-19; PerS = perceived severity; PerC = perceived controllability. Neg. = negative emotion; Pos. = positive emotion; Sleep = sleep problems; Agg. = aggression; SU = substance use; MPU = mobile phone use; Soc. P. = social participation; Pre. B = precautionary behaviour. Unstandardized coefficients are reported. Solid lines represent significant coefficients; dash lines represent non-significant coefficients. The effects of covariates on the outcomes are omitted for simplicity; specific effects could be found in Table 5. *p < .05; ** *p* < .01; *** *p* < .001

The model fit was acceptable, *χ*^2^(655) = 6502.36, *RMSEA* = .044, *CFI* = .935, *TLI* = .921. Results are summarized in Fig. 1 and Table 5.

**Table 5:** The association between knowledge, perceived severity, perceived controllability about the COVID-19 and emotional and behavioural reactions, social participation, and precautionary behaviour

	Neg.		Pos.		Sleep.		Agg.		SU		MPU		Soc. P.		Pre. B	
	*B*	*S.E.*	*B*	*S.E.*	*B*	*S.E.*	*B*	*S.E.*	*B*	*S.E.*	*B*	*S.E.*	*B*	*S.E.*	*B*	*S.E.*
Sex	.06^*^	.02	.06^*^	.03	−.02	.03	−.02	.03	−.06^*^	.03	.07^*^	.04	−.04	.03	.19^***^	.02
Age	.00	.00	.01^**^	.00	.00	.00	−.01^**^	.00	−.01^**^	.00	−.01^*^	.00	.01^***^	.00	−.01^***^	.00
Phy.history	−.01	.04	.02	.05	−.01	.05	−.06	.05	−.02	.05	−.15^*^	.07	.10	.06	−.07^*^	.04
Psy.history	−.02	.11	.08	.15	−.41^***^	.12	−.04	.14	−.07	.13	−.05	.16	−.03	.15	.13	.11
Health con.	−.13^***^	.01	.01	.02	−.14^***^	.02	−.10^***^	.02	−.07^***^	.02	−.11^***^	.02	.10^***^	.02	.12^***^	.01
Education	.07^***^	.01	−.04^*^	.02	.08^***^	.02	.10^***^	.02	.10^***^	.02	.08^***^	.02	.07^***^	.02	.07^***^	.01
Rel. w. COVID-19	−.00	.07	.04	.08	−.03	.09	−.02	.08	−.09	.08	.09	.10	−.05	.08	−.06	.08
PerS	.34^***^	.03	.22^***^	.03	.10^***^	.03	.01	.03	−.01	.03	.47^***^	.04	−.04	.03	.36^***^	.03
PerC	−.06^***^	.02	−.08^***^	.02	−.04	.02	−.00	.02	.01	.02	−.00	.03	.05^*^	.02	.09^***^	.02
Know	−.01	.02	−.03	.03	.03	.03	.00	.03	.00	.03	−.02	.03	.28^***^	.03	.24^***^	.02
*R*^2^	11.1%^***^		3.3%^***^		3.3%^***^		2.8%^***^		1.9%^***^		7.1%^***^		6.8%^***^		22.5%^***^	

*Note*. *Phy.history* self-reported history of chronic physical diseases, *Psy.history* self-reported history of psychiatric / psychological disorder, *Health con.* self-reported current physical health condition, *Rel. w. COVID-19* = relationship with the COVID-19, *PerS* = perceived severity; *PerC* perceived controllability, *Know* knowledge about the COVID-19, *Neg.* negative emotion, *Pos.* positive emotion, *Sleep* sleep problems, *Agg.* aggression, *SU* substance use, *MPU* mobile phone use, *Soc. P.* social participation; *Pre. B* precautionary behaviour. *p < .05; ** *p* < .01; *** *p* < .001

The model explained 11.1% variance of the changes in negative emotion. The results showed that being female, having higher levels of education, and perceiving the virus to be more severe were related to more increase in negative emotion since the outbreak of COVID-19. In contrast, having better physical health condition and perceiving the virus to be controllable were related to less increase in negative emotion.

The model explained 3.3% variance of the changes in positive emotion. The results showed that being female, being older and perceiving the virus to be severe were related to more decrease in positive emotion since the outbreak of COVID-19. In contrast, having higher levels of education and perceiving the virus to be controllable were related to less decrease in positive emotion.

The model explained 3.3% variance of the changes in sleep problems. The results showed that having higher levels of education and perceiving the virus to be more severe were related to more increase in sleep problems since the outbreak of COVID-19. In contrast, being without history of psychiatric/psychological disorder and having good physical health condition were related to less increase in sleep problems.

The model explained 2.8% variance of the changes in aggression. The results showed that having higher levels of education was related to more increase in aggression since the outbreak of COVID-19. In contrast, being older and having good physical health condition were related to less increase in aggression.

The model explained 1.9% variance of the changes in substance use. The results showed that having higher levels of education was related to more increase in substance use since the outbreak of COVID-19. In contrast, being female, being older and having good physical health condition were related to less increase in substance use.

The model explained 7.1% variance of the changes in mobile phone use. The results showed that being female, having higher levels of education and perceiving the virus to be more severe were related to more increase in mobile phone use since the outbreak of COVID-19. In contrast, being younger, being without history of physical chronic disease, and having better physical health condition were related to less increase in mobile phone use.

The model explained 6.8% variance of social participation. The results showed that being older, having better physical health condition, having higher levels of education, perceiving the virus to be more controllable, and having more knowledge about the virus were related to more social participation since the outbreak of COVID-19.

The model explained 22.5% variance of precautionary behaviour. The results showed that being female, having better physical health condition, having higher levels of education, perceiving the virus to be more severe and controllable, and having more knowledge about the virus were related to more precautionary behaviour since the outbreak of COVID-19. In contrast, being younger and being with history of chronic physical diseases were related to less precautionary behaviour.

## Discussion

### The public’s emotional and behavioural reactions, social participation, and precautionary behaviour during the outbreak of COVID-19

A central goal of this research is to provide early evidence to the understanding of public’s emotional and behavioural outcomes during the outbreak of COVID-19. In this study, we examined three related outcomes, including the changes in the frequencies of the public’s emotional and behavioural reactions towards COVID-19 before and after the outbreak, the public’s participation in social events regarding COVID-19, and the public’s engagement in precautionary behaviour. Regarding the changes in the frequencies of the public’s emotional and behavioural reactions, participants reported very slight changes in experiencing negative emotion, positive emotion and using mobile phone before and after the outbreak of COVID-19, as the mean score of these dimensions were higher than 3 (coded *as more or less the same before and after the outbreak of COVID-19*) but less than 4 (coded *higher compared to the days before the outbreak*). Interestingly, compared to the days before the outbreak, the public reported slightly fewer sleep problems, less aggression and substance use after the outbreak. These finding suggest that the outbreak of COVID-19 does not necessarily bring intensive negative emotional or behavioural responses; on the contrary, it may also bring slight benefit, such as showing less aggression, drinking, smoking, and fewer sleeping problems. These results were not consistent with prior studies which found that citizen showed a number of mental health problems due to the outbreak of COVID-19 [20, 21]. In addition, the low levels of social participation and the high levels of precautionary behaviour suggest that the public did not show too much interest in participating in social events but that they developed a good habit of behaviour that prevent the virus in time of the. The high levels of preventive behaviour found in this study are largely consistent with most studies which disclosed that citizens had strong intention to engage in preventive behaviour [18, 19].

These reactions could be due to several reasons. First, they may be related to the measures the Chinese government has adopted since the outbreak in controlling the transmission of COVID-19, including urging the public to maximally stay at home and reduce mobility, suspending most public facilities and venues that could be crowded (e.g., bars, cinema, restaurants, etc.), strengthening the monitoring of physical health whenever the citizen enter the public venue (e.g., supermarkets, ones’ residential building), issuing precautionary guidance to maintain good mental and physical health, and strongly urging the public to keep personal hygiene. Such strong measures and the transparency of the media may increase the public’s mental and intellectual resource to maintain their mental and physical health, which thus restrains the deterioration of emotional and behavioural responses and increases healthy habits. However, staying at home for a long time could also increase the frequency of using mobile phone to maintain social connection, work from home, and reduce boredom. Second, the low levels of social participation could be because the stay-at-home and social distancing policies and thus most participants just lived their lives and limited the extent to which they engaged in social events. Another reason might be because most of the participants in this study were students and they had limited capacity to actually contribute to the control of COVID-19. Third, the timing of the outbreak of COVID-19 might also matter. Actually, the outbreak of COVID-19 was reported just right before the Chinese New Year, the time for most citizens to get back hometown and gather with family. This allows most citizens to stay with family and support each other; strong social support is crucial resource to alleviate stress caused by natural disaster or induced by experimental manipulation and to maintain physical and mental health [35, 36].

### The role of cognitive appraisal in the emotional and behavioural reactions towards the outbreak of COVID-19

Three cognitive appraisals were examined. The results suggested that the public had differential evaluation regarding these appraisals. These findings align with prior researches that reveal the differences in the levels of various cognitive appraisals in the research of mental health and well-being [3, 12, 13, 17]. Besides the differences in the mean levels, these appraisals were associated with the outcomes differentially. Among the three appraisals, perceived severity was the risk factor most widely associated with emotional and behavioural reactions, which is consistent with prior studies [22–25]. In addition, we found that perceived controllability was the protective factor against the emotional problems. However, knowledge about COVID-19 was not related to any emotional and behavioural reactions, which is not consistent with prior studies [25, 26]. This may be because evaluation of severity is more closely about whether and how much an individual’s health and well-being is at stake compared to the other two types of appraisals. This suggests that different cognitive appraisals are differentially related to emotional and behavioural outcomes, as found in previous studies [13, 37, 38]. Another explanation would be that because knowledge about COVID-19 was associated with the other two cognitive appraisal factors (*r* = .13 with perceived severity and *r* = .37 with perceived controllability), its association with the emotional and behavioural outcomes would be reduced once the other two appraisal factors were controlled for. This highlights the importance of taking different cognitive appraisal factors into account when examining their respective associations with the outcomes.

Moreover, all the three appraisals were positively related to more social participation (except for perceived severity) and precautionary behaviour. These findings are consistent with previous studies that cognitive appraisal (e.g., having more knowledge and perceived risk, threat and danger) were related to more precautionary behaviour during the outbreak of SARS [3, 17, 39] and COVID-19 [25]. In addition, these findings suggest that although perceived severity of COVID-19 is generally related to more emotional and behavioural problems, it is related to more precautionary behaviour ------ actions that help control the epidemics [5]. In this sense, perceived severity can be regarded as a double-edge sword, being both risk and asset, in the encounter of medical catastrophe.

### Implications

This study has several theoretical and practical implications. Theoretically, the findings deepen our understanding of the important role of cognitive appraisal in the emotional and behavioural outcomes during the outbreak of COVID-19. Specifically, the findings contribute to the literature that some cognitive appraisals (e.g., know more information and perceived controllability of the event) may serve as protective factors for some emotional and behavioural outcomes. Moreover, some commonly believed risk factor such as perceived severity may actually entail beneficial effect to some extent, such as associating with more behaviour that help control the epidemic (e.g., precautionary behaviour). Taken together, these findings highlight the utility of cognitive appraisal in the explanation of the public’s emotional and behavioural responses towards emergent public health concern. Nevertheless, it should be noted that other than negative emotion and precautionary behaviour, the three types of cognitive appraisal factors only accounted for a small proportion of variance in some outcomes, particular for substance use and aggression. This suggests that future research may need to explore other predictors of these problems.

Practically, the findings bear several implications for policy-makers and frontline practitioners. First, we identified some groups that are generally vulnerable to various emotional and behavioural problems, such as being female, having physical health problems concurrently, and having higher education level. Therefore, these groups may be in higher need of mental care. Second, the public’s cognitive appraisals are related to different outcomes. This suggests that practitioners may address different emotional and behavioural problems by intervening with relevant cognitive appraisals. For instance, alleviating the public’s perceived severity of COVID-19 and increasing their perceived controllability might be a promising way to reduce negative emotion after the outbreak. Third, policy-makers and governments should publicize scientific information about the virus to the public as thorough and detailed as possible, since this might enhance the public’s motivation engaging in behaviour that might help control the epidemic (e.g., social participation and precautionary behaviour).

### Limitations

This study has several limitations we must acknowledge. First, this study relied on self-report data and cross-sectional design. The cross-sectional design precludes causal inferences as well as assumptions about the direction of causality. Moreover, although common method bias did not appear to be a concern in this study, its potential effect could not be excluded. However, this is not without precedent when examining the public’s emotion and behaviour during the outbreak of virus/disease [3, 4, 17]. As a preliminary study, the findings provide early understanding of the public’s cognitive appraisals of COVID-19 and their association with a number of emotional and behavioural outcomes. Nevertheless, future study may utilize more sophisticated design and multiple-informant to achieve more robust results. Second, we adopted a number of measures to maximize that the current sample was valid (e.g., we removed participants with a similar responding pattern). However, we must acknowledge that the current sample is not representative of the Chinese population. Females, participants with high education, and participants residing in Guangdong Province were over-representative in the sample, which may limit the generalizability of the findings. Third, we developed measures specific to the outbreak of COVID-19 in this study. A number of analyses support the reliability and validity of the measures used in this study, but we must acknowledge that these measures could be enhanced. For instance, in the confirmatory factor analyses of some measures (e.g., precautionary behaviour), acceptable model fit could be obtained only when some residuals are correlated probably due to similar meaning of the items. Given the unpredictable development of COVID-19 around the globe at the moment, it would be desirable for scholars to refine the existing instruments or to develop new instruments to better examine people’s psychological and behavioural reactions towards COVID-19. Fourth, with Harman’s single-factor test, we did not find common method variance to be a severe issue in this study. However, Harman’s single-factor test is a diagnostic rather than a remedial approach and has been severely criticized [40]. Therefore, we must acknowledge that readers should interpret the results with caution. Finally, we need to emphasize again that the model explained only small amount of variance in many outcome variables, suggesting that relying on cognitive appraisal to understand the public’s emotional and behavioural outcomes are not enough and future research should investigate other relevant predictors as well.

## Conclusions

When the current study was conducted, China was taking enormous efforts to control COVID-19. To this end, intellectual input from multiple disciplines is required, including the understanding of the public’s emotion and behaviour and their antecedents from the psychological perspectives. This study provided early evidence to this issue at the early stage of the outbreak of COVID-19. Our results revealed that the public’ emotional and behavioural problems before and after the outbreak of COVID-19 did not change too much. The public had limited participation in social events regarding COVID-19 but they actively engaged in precautionary behaviour. Moreover, the public’s appraisals (i.e., knowledge, perceived severity and perceived controllability) of COVID-19 were differentially related to their emotional and behavioural outcomes. We believe that these findings bear important theoretical and practical implications in understanding the public’s emotion and behaviour during the outbreak of COVID-19. Besides, now that the control of the epidemic has become a regular routine, it is necessary to exert continuous efforts to track the public’s emotional and behavioural reactions and to enhance their well-being during the current regular anti-COVID-19 period.

## Supplementary information

**Additional file 1.**

## Data Availability

The measures are attached as supplementary files and the dataset is available from the corresponding authors upon request.

## Abbreviations

CFI: Comparative Fit Index
COVID-19: Coronavirus Disease-19
RMSEA: Root-Mean-Square Error of Approximation
SARS: Severe Acute Respiratory Syndrome
TLI: Tucker-Lewis Index

## Footnotes

1 Because the online survey website automatically set each question as “required to be answered”, participants who undertook the study needed to finish the survey prior to submission. Participants who did not want to continue could quit the survey by closing the window. However, the online survey website did not record the response of participants who quitted the survey in the middle and therefore participants included in the analysis were the ones who provided complete data.

2 Measures used in this study can be found in online supplementary files.
