# Supplementary material for: Chinese public’s knowledge, perceived severity, and perceived controllability of COVID-19 and their associations with emotional and behavioural reactions, social participation, and precautionary behaviour: a national survey
Source: BMC Public Health. 2020 Oct 21;20:1589. doi: 10.1186/s12889-020-09695-1 (PMC7576982; doi:10.1186/s12889-020-09695-1)
Supplement: Supplementary file 1 — Additional file 1. [file 12889_2020_9695_MOESM1_ESM.docx]

**Online supplementary files**

**Emotional and behavioral reactions**

Please compare with the days before COVID-19 outbreak to indicate how much your emotions and behaviors have changed after the outbreak.

Keys:

1 = much less compared to the days before the outbreak

2 = less compared to the days before the outbreak

3 = almost the same compared to the days before the outbreak

4 = more compared to the days before the outbreak

5 = much more compared to the days before the outbreak

1. Feel anxious
2. Feel worried
3. Feel depressed
4. Feel panic
5. Feel lonely
6. Feel nervous
7. Feel sad
8. Feel angry
9. Feel happy
10. Feel joyful
11. Feel excited
12. Have insomnia
13. Have shallow sleep
14. Have nightmare
15. Have insufficient sleep
16. Argue with others (family, friends, colleagues, strangers, etc.)
17. Fight with others (family, friends, colleagues, strangers, etc.)
18. Drink alcohol
19. Smoke cigarette
20. Use mobile phone

**Social participation**

Since COVID-19 outbreak, how often do you engage in the following behaviors?

Keys:

1 = never

2= seldom

3= sometimes

4= often

5= very often

1. Help others increase their life quality
2. Help those who need help in the community
3. Dedicate time, donate money or supplies to chartered organizations or relevant institutes (e.g., hospitals)
4. Help others master the knowledge and ways to prevent COVID-19
5. Discuss COVID-19 with others.

**Precautionary behavior**

Since COVID-19 outbreak, how often have you engaged in the behaviors listed below?

Keys:

1 = never

2= seldom

3= sometimes

4= often

5= very often

1. Avoid heading to the infected regions.
2. Wear a face mask.
3. Change a face mask regularly.
4. Deal with the used face mask appropriately (e.g., pick off the face mask from the side, put it in the garbage bin and disinfect it, etc.)
5. Avoid gathering.
6. Wash hands.
7. Use disinfectant.
8. Pay attention to personal hygiene.
9. Avoid contacting with a certain groups of population.
10. Pay attention to balanced diet.
11. Avoid going to the public.
12. Stay at home as much as possible.
13. Avoid eating outside.
14. Avoid using public facilities.
15. Avoid using public transportation.
16. Get sufficient sleep.
17. Closely monitor personal physical health.
18. Closely monitor the physical health of the people around you.
19. Persuade the people around you to following the government’s precautionary guidance.

**Knowledge of COVID-19**

The following items ask how much you know about COVID-19. Please choose the option that most indicates your opinion/thoughts/situation, according to your understanding.

Keys:

1 = totally not clear

2 = not clear

3 = neutral

4 = clear

5 = totally clear

1. Its etiology
2. Ways of transmission
3. Infectiousness
4. Symptoms
5. Diagnostic criteria
6. Ways of treatment
7. Recovery criteria
8. Rate of recovery
9. Infectiousness after recovery
10. The mortality of the confirmed cases
11. Preventive approaches

**Perceived severity**

The following items ask you about how severe you think of COVID-19. Please choose the option that most indicates your opinion/thoughts/situation, according to your understanding.

Keys:

1 = not severe at all;

2 = not severe

3 = neutral

4 = severe

5 = very severe

1. How severe you think of the infectiousness of COVID-19?
2. How severe you think of the morbidity of COVID-19?
3. How severe you think of the mortality of COVID-19?
4. How severe you think of the impact of COVID-19 on the social order?
5. How severe you think of the impact of COVID-19 on the economy of the society?

**Perceived controllability**

The following items ask you about how controllable you think of COVID-19. Please choose the option that most indicates your opinion/thoughts/situation, according to your understanding.

Keys:

1 = totally uncontrollable;

2 = uncontrollable;

3 = neutral

4 = controllable

1. = totally controllable
2. The etiology
3. The ways of transmission
4. The infectiousness
5. The time COVID-19 will last
6. The effectiveness of the treatment
7. The recovery rate
8. The mortality
9. The developmental trends of COVID-19
10. The overall controllability of COVID-19
